# Dengue, Chikungunya, and Zika: Spatial and Temporal Distribution in Rio de Janeiro State, 2015–2019

**DOI:** 10.3390/tropicalmed7070141

**Published:** 2022-07-20

**Authors:** Paula Maria Pereira de Almeida, Aline Araújo Nobre, Daniel Cardoso Portela Câmara, Luciana Moura Martins Costa, Izabel Cristina dos Reis, Mário Sérgio Ribeiro, Cristina Maria Giordano Dias, Tania Ayllón, Nildimar Alves Honório

**Affiliations:** 1 Laboratório de Mosquitos Transmissores de Hematozoários—Lathema, Instituto Oswaldo Cruz/Fiocruz, Rio de Janeiro 21040-900, Brazil; dcpcamara@gmail.com (D.C.P.C.); izabel.cristina@ioc.fiocruz.br (I.C.d.R.); tayllon@ucm.es (T.A.); 2 Núcleo Operacional Sentinela de Mosquitos Vetores—Nosmove/Fiocruz, Rio de Janeiro 21040-900, Brazil; 3 Secretaria de Estado de Saúde do Rio de Janeiro—SES/RJ, Rio de Janeiro 20031-142, Brazil; mario.ribeiro@saude.rj.gov.br (M.S.R.); crismgd10@gmail.com (C.M.G.D.); 4 Programa de Computação Científica da Fiocruz-PROCC/Fiocruz, Rio de Janeiro 21040-900, Brazil; aline.nobre@fiocruz.br (A.A.N.); luciana.costa@posgrad.ensp.fiocruz.br (L.M.M.C.); 5 Department of Genetics, Physiology and Microbiology, Faculty of Biological Sciences, University Complutense of Madrid, 28040 Madrid, Spain

**Keywords:** arboviruses, *Aedes* spp., spatial and temporal dynamics, Rio de Janeiro

## Abstract

Simultaneous spatial circulation of urban arboviral diseases, such as dengue, chikungunya, and Zika, is a major challenge. In this ecological study of urban arboviruses performed from 2015 to 2019, we analyzed the spatial and temporal dynamics of these arboviruses in all 92 municipalities and nine health regions of Rio de Janeiro state. Annual cumulative incidences are presented for all three arboviruses throughout the study period. Spatial analyses of the three studied arboviruses showed distinct behaviors among municipalities and health regions. Co-circulation of the three arboviruses in the state and a heterogeneous spatiotemporal pattern was observed for each disease and region, with dengue having a higher annual incidence during the five years of the study, as well as two consecutive epidemic years in the state. The increase in transmission in different regions of the state in one year culminated in an epidemic in the state in the following year. A high annual cumulative incidence of chikungunya occurred in municipalities from 2017 to 2019 and of Zika only in 2016. Some municipalities with higher population densities showed higher incidences for some arboviruses and appeared to contribute to the dissemination to cities of lower demographic density and maintenance of these urban arboviruses. Thus, regions recording increased incidences of the three diseases in their territories for long periods should be considered municipal poles, as they initiated and sustained high transmission within their region.

## 1. Introduction

Urban arboviruses, such as dengue, chikungunya, and Zika, are emerging and re-emerging infectious diseases and serious global public health problems [1,2,3,4] that are transmitted by mosquitoes of the genus *Aedes*. Culicidae are among the arthropod vectors that are the predominant transmitters of these arboviruses to humans, and their epidemic cycle involves transmission between humans by infected females, particularly *Aedes aegypti* (L.) and *Ae. albopictus* (Skuse) [3,5].

The growing incidence and geographic expansion of these arboviruses suggest limitations for current surveillance and vector control strategies that require intersectoral actions from health authorities [2,3]. Dengue prevails among re-emerging diseases as an important arthropod-borne viral disease. In Brazil, dengue virus (serotypes DENV-1 and DENV-4) was first recognized in Roraima, in the northern region of the country in 1981–1982 [6]. Since the first report, dengue has spread across the country, causing several epidemics, with the first major epidemic described in the state of Rio de Janeiro in 1986 [7]. The state of Rio de Janeiro was the entry point for dengue serotypes DENV-2 and DENV-3, which caused epidemics in 1990–1991 and 2001–2002, respectively. In 2008, the state experienced another epidemic, caused by DENV-2, which proved to be more severe than previous episodes [8,9,10]. In contrast, chikungunya was first documented in Brazil in 2014 in cities in the states of Amapá and Bahia. Although chikungunya causes more intense symptoms of arthralgia, it features a clinical picture similar to that of dengue, increasing the likelihood of misdiagnosis between the two diseases [2]. Finally, the Zika virus was discovered decades ago circulating endemically mainly in Africa, and it emerged recently in the Pacific Ocean islands until it reached South America and Brazil in 2015 [4]. Zika was first detected in Brazil in 2015; however, it was subsequently determined to have been introduced in 2013, probably from French Polynesia or Easter Island [4]. Evidence of the early introduction of ZIKV in Brazil was revealed by phylogenetic analysis of infected mosquitoes collected in densely populated low-income areas in Rio de Janeiro [11]. The initial Zika outbreak occurred in the state of Bahia. Due to similar clinical signs and cross-reactivity, it was originally believed to be the dengue virus [12]. Zika quickly became a serious public health problem due to its association with microcephaly in newborns, and it was declared a public health emergency of national concern (PHENC) in Brazil in 2015 and a public health emergency of international concern (PHEIC) by the World Health Organization (WHO) in 2016 [4,13,14].

Recent data have revealed 971,136 notified cases of dengue reported in Brazil in 2020 (462.1 cases per 100,000 inhabitants). In the same year, 78,808 notified chikungunya cases (37.5 cases per 100,000 inhabitants), and 7006 notified Zika cases were reported in the country (3.3 cases/100,000 inhabitants). From January to October 2021, 477,209 notified cases (223.7 cases per 100,000 inhabitants) of dengue were reported in Brazil, representing a reduction of 47.8% in registered cases for the same analyzed period of 2020. For chikungunya, 85,794 notified cases were reported (40.2 cases per 100,000 inhabitants), corresponding to an increase of 27.6% compared to those in the previous year. There were 5361 notified cases (2.5 cases per 100,000 inhabitants) of Zika reported, representing a 19.4% reduction in the number of cases in the country in relation to those in the same period in 2020.

The Rio de Janeiro state in Brazil has the third largest population, over 17 million people, and the second highest population density in the country (365 inhabitants per square kilometer) [15,16]. Densely urbanized and endemic areas are ideal for the simultaneous spatial circulation of different arboviruses, which is a major challenge to public health. This is the case in Rio de Janeiro because of the sociodemographic and environmental conditions that favor the dispersal of mosquito vectors and viruses [17,18]. Moreover, the dynamics of urban arboviruses are seasonal, as has been shown in several endemic areas of the world, with greater transmission reported during the summer months [19].

While multiple studies have evaluated the spatial and temporal distribution of arboviruses in the state of Rio de Janeiro, they have generally focused on one arbovirus without investigating the triple endemicity in the entire state. Several ecological and economic factors, such as those in Rio de Janeiro, may contribute to the transmission of arboviruses. Therefore, a deeper study of these three arboviruses is necessary to gain a better understanding of the dynamics of their spread over time and space in vulnerable areas [18,20]. The present study aimed to analyze the spatial and temporal dynamics of the simultaneous circulation of dengue, chikungunya, and Zika in the state of Rio de Janeiro between 2015 and 2019, considering 92 municipalities distributed across nine health regions of the state.

## 2. Material and Methods

### 2.1. Study Design

This was an ecological study of notified reported cases of dengue, chikungunya, and Zika in the state of Rio de Janeiro from 2015 to 2019. The 92 municipalities distributed in nine health regions constituted the units of spatial analysis, and months and years were the temporal units of the studied period. Notified cases corresponded to those with suspected cases reported, except those that were discarded according to the definition of the Ministry of Health.

#### Ethics Committee

The present project was approved by the Research Ethics Committee of the Oswaldo Cruz Institute under CAAE 35291720.1.0000.5248.

### 2.2. Study Area

**Figure 1:**
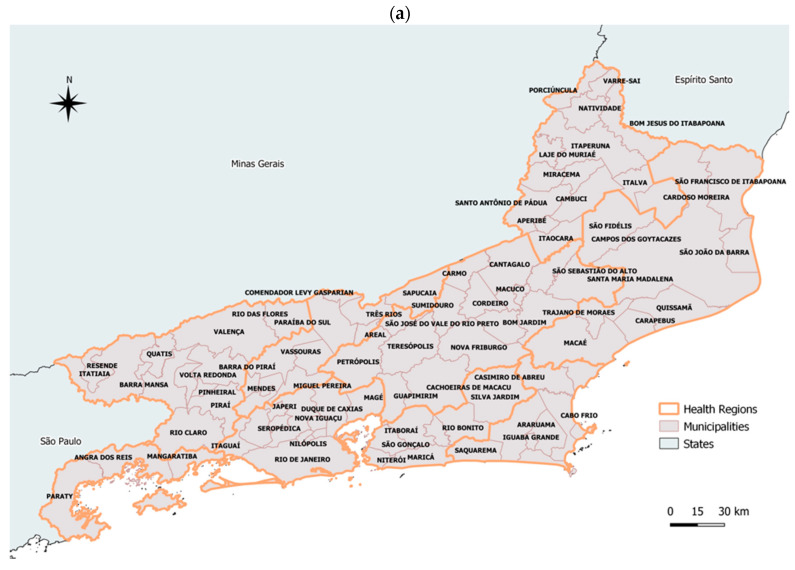

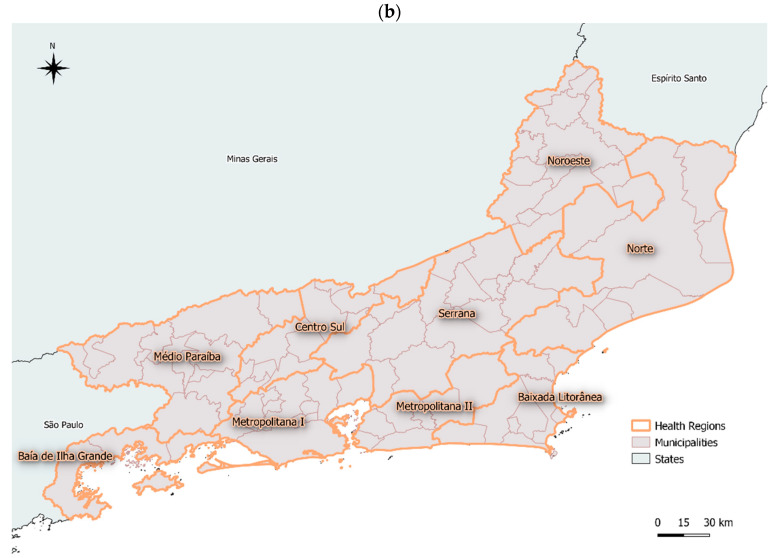
Map of the Rio de Janeiro state and its municipalities (**a**) and nine health regions (**b**). Source: Own elaboration.

The state of Rio de Janeiro is located on the eastern side of southeastern Brazil. It has an estimated population of 17,366,189 with an area of 43,777.954 km^2^, delimited by the states of Minas Gerais, Espírito Santo, and São Paulo, and the Atlantic Ocean. The state consists of 92 municipalities (Figure 1a) and is part of the Atlantic Forest biome, presen-ting diversified landscapes with mountains and lowlands along with sandbanks, bays, lagoons, and tropical forests. In 2009, the Rio de Janeiro state finalized the distinction of health regions along with the regional planning process, creating a total of nine regions: Baía de Ilha Grande, Centro-Sul, Baixada Litorânea, Metropolitana I, Metropolitana II, Médio Paraíba, Noroeste, Norte, and Serrana (Figure 1b). Each region was aggregated according to contiguous municipalities that have similarities, with a health surveillance center (Núcleo de Vigilância em Saúde—NDVS) acting in each region as an administrative unit of the State Health Secretariat of Rio de Janeiro.

### 2.3. Data Source

Notified reported cases of dengue, chikungunya, and Zika were obtained from the Notifiable Diseases Information System (SINAN) bank of the Rio de Janeiro State Department of Health (SES/RJ) for the period from 2015 to 2019, considering the date of symptom onset. The estimated population for each study period and for each municipality and health region was obtained from the Brazilian Institute of Geography and Statistics website (https://cidades.ibge.gov.br/, accessed on 21 May 2021).

### 2.4. Statistical Analysis

For statistical purposes, notified reported cases of dengue, chikungunya, and Zika were aggregated into three and two different spatial and temporal units, respectively. Summary measures of annual cumulative incidence were calculated for each arbovirus considering the spatial scale of the state and municipalities. Time series graphs were constructed to evaluate the behavior of the monthly incidence rate of each arbovirus throughout the study period, considering the state and nine health regions as units of spatial analysis. To evaluate seasonality, boxplots were constructed monthly for each arbovirus. Thematic maps of the accumulated incidence of cases per year and municipality were created for the nine health regions. QGIS (version 3.12.3) and R (version 4.0.0) were used for elaboration of the maps and summary statistics and graphics, respectively.

## 3. Results

During the study period (2015–2019), 439,607 notified cases of urban arboviruses were reported in the Rio de Janeiro state, including 204,719 dengue cases (annual cumulative incidence of 182.6 cases/100,000 inhabitants), with 2016 having the highest number (84,768 cases reported and an incidence of 509.5/100,000 inhabitants). Chikungunya returned 146,302 cases (annual cumulative incidence of 167.9/100,000 population), with the highest occurrence in 2019 (84,567 cases and an incidence of 492.8/100,000 population). Zika was reported in 88,586 cases (annual cumulative incidence of 15.3/100,000 population), with the highest occurrence in 2016 (71,692 cases and an incidence of 430.9/100,000 population).

In the state, the definition of epidemic years considered the value of the average annual incidence above 300 cases/100,000 inhabitants for each arbovirus, according to the following criteria established by the Ministry of Health to dengue: up to 100 cases/100,000 inhabitants was considered low incidence, between 100 and 300 cases/100,000 inhabitants was considered medium incidence, and above 300 cases/100,000 inhabitants, high incidence [21]. The epidemics caused by the three arboviruses reported in the state of Rio de Janeiro occurred in 2015 and 2016 for dengue, 2019 for chikungunya, and 2016 for Zika (Table 1).

Table 1 shows the averages and medians of the annual cumulative incidences in Rio de Janeiro state obtained from the values of the 92 municipalities. A large variation among the incidences and municipalities was observed when analyzing the minimum and maximum values. In 2016, the highest number of municipalities had an incidence of > 300 cases/100,000 inhabitants for dengue (54 municipalities or 58.7% of the municipalities in the state). Dengue was present with a high incidence (>300 cases/100,000 inhabitants) throughout all years, affecting 116 municipalities, while chikungunya registered high incidences in 2017 (one municipality), 2018 (12 municipalities; 13.0%), 2019 (38 municipalities; 41.3%), and Zika had a high incidence in 2016 (25 municipalities; 27.2%).

**Figure 2:**
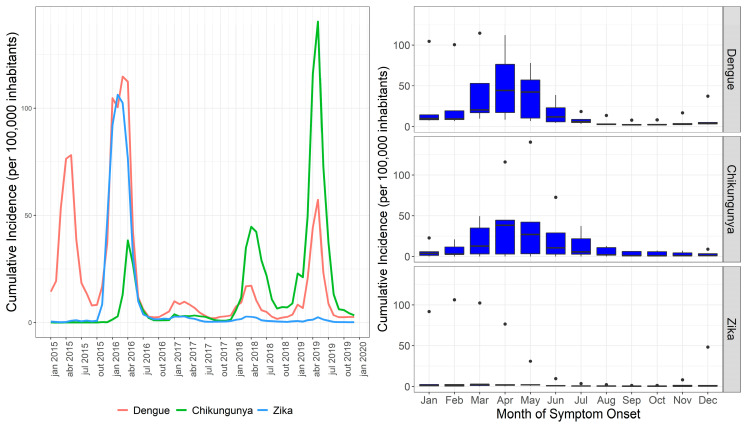
Dengue, chikungunya, and Zika, by month of symptom onset, Rio de Janeiro state, 2015–2019.

Temporal analysis of the three urban arboviruses in the state of Rio de Janeiro highlighted a distinct pattern (Figure 2). The monthly incidence rate of dengue peaked on the transmission curve in May 2015 (78.2 cases/100,000 inhabitants). In 2016, dengue had a monthly incidence of >100 cases/100,000 inhabitants in January, peaking in April 2016 (112.4 cases/100,000 inhabitants).

Chikungunya and Zika reached their transmission peaks in May 2019 (140.4 cases/100,000 individuals) and February 2016 (106.3 cases/100,000 individuals), respectively. In 2016, dengue and Zika showed similar transmission curves; however, the dengue peak was observed after (in April) that of Zika (in February). Dengue showed the highest average annual cumulative incidence (>1000 cases/100,000 inhabitants) and the greatest range (municipalities with annual incidences between 5 and 9000 cases/100,000 inhabitants) in 2016, and municipalities were recorded with high incidence over the five years.

Chikungunya did not show a high incidence in municipalities in 2015 and 2016, while Zika produced epidemic situations in municipalities in 2016 (Figure 2 and Table 1). However, it is observed that chikungunya started to increase transmission in April 2016, while Zika increased in October 2015 (Figure 2).

The spatial and temporal distributions of dengue, chikungunya, and Zika in the state of Rio de Janeiro were assessed and detailed in the following sections.

### 3.1. Dengue Spatial and Temporal Distribution

**Figure 3:**
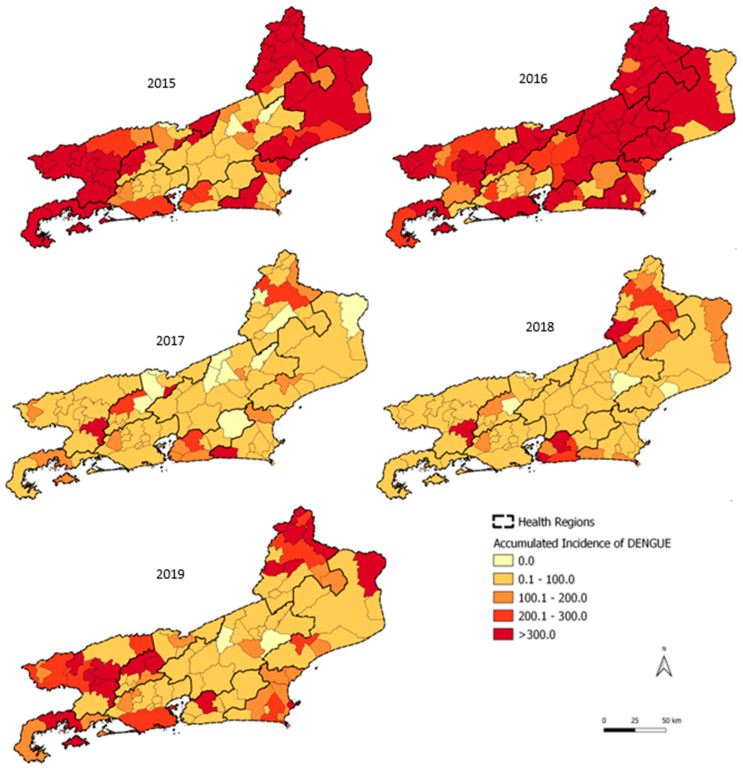
Dengue by year of symptom onset in the state of Rio de Janeiro, 2015–2019.

**Figure 4:**
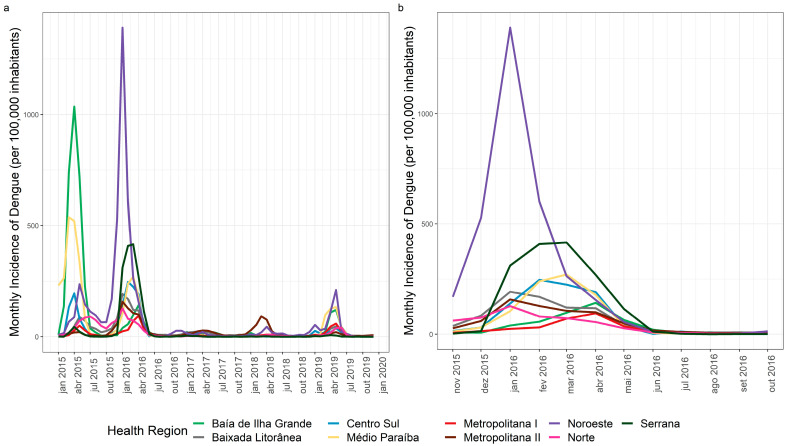
(**a**) Dengue by month/year of symptom onset in Rio de Janeiro state, 2015–2019. (**b**) Dengue by month/year of symptom onset in Rio de Janeiro state, November 2015–October 2016.

The Norte and Noroeste regions sustained annual incidences of >300 cases/100,000 inhabitants in 2015 and 2016. In 2019, there was no dengue epidemic in the state; however, some municipalities in Noroeste, Médio Paraíba, and Baía de Ilha Grande reached a high annual incidence: Angra dos Reis in Baía de Ilha Grande, Volta Redonda, Barra do Piraí, Pinheiral, Piraí, and Quatis in Médio Paraíba; and Porciúncula, Natividade, Bom Jesus de Itabapoana, Miracema, and São José de Ubá in Noroeste (Figure 3). In 2015, the health regions with the highest monthly incidence rates were Baía de Ilha Grande (1035.5 cases/100,000 inhabitants) in April and Médio Paraíba (537.8 cases/100,000 inhabitants) in March. In 2016, the highest incidences were recorded in the Noroeste regions (1390.5 cases/100,000 inhabitants) in January and in the Serrana region (415.7 cases/100,000 inhabitants) in March (Figure 4a). Temporal analysis of the monthly epidemiological curve of dengue in the health regions from November 2015 to October 2016 returned higher peaks in January 2016, especially in the Baixada Litorânea, Metropolitana II, Norte, and Noroeste regions. Peaks occurred later in Centro-Sul (February), Médio Paraíba and Serrana (March), and Baía de Ilha Grande and Metropolitana I (April 2016; Figure 4b).

### 3.2. Chikungunya Spatial and Temporal Distribution

**Figure 5:**
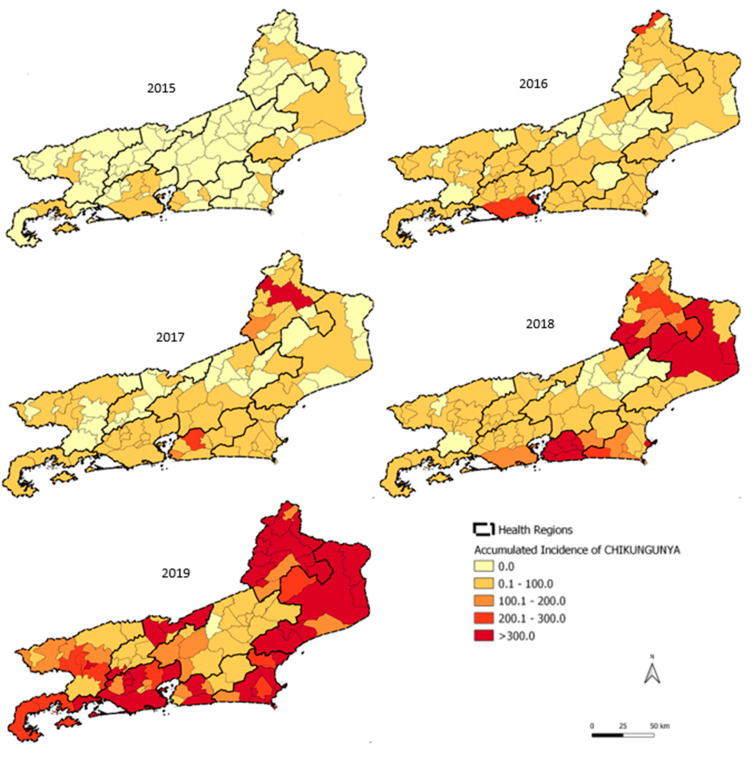
Chikungunya by year of symptom onset in the state of Rio de Janeiro, 2015–2019.

**Figure 6:**
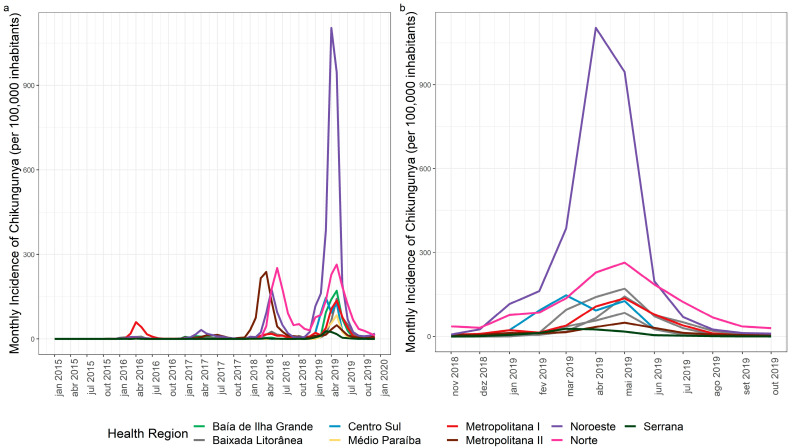
(**a**) Chikungunya by month/year of symptom onset in Rio de Janeiro state, 2015–2019. (**b**) Chikungunya by month/year of symptom onset in Rio de Janeiro state, November 2018–October 2019.

The municipalities of Itaboraí (Metropolitana II), Campos dos Goytacazes (Norte region), and Santo Antônio de Pádua (Noroeste region) maintained an annual incidence of above 300 cases/100,000 inhabitants in 2018 and 2019, with records of high transmission for two consecutive years (Figure 5). The Metropolitana I region had the highest monthly incidence rate of chikungunya in April 2016 (60.2 cases/100,000 inhabitants), highlighting the city of Rio de Janeiro. In 2018, the highest monthly incidences were recorded in the Norte region (251.7 cases/100,000 inhabitants) in June, the Metropolitana II region (238.1 cases/100,000 inhabitants) in April, and the Noroeste region (175.8 cases/100,000 inhabitants) in May. In 2019, which was an epidemic year for the state of Rio de Janeiro, the highest incidence was recorded in the Noroeste region (385.9 cases/100,000 inhabitants) in March and the Norte region (264.2 cases/100,000 inhabitants) in May (Figure 6a). The Centro-Sul and Noroeste regions reached higher peaks in March and April 2019, respectively, differing from the other regions of the state, which showed later peaks in transmission in May 2019 (Figure 6b).

### 3.3. Zika Spatial and Temporal Distribution

**Figure 7:**
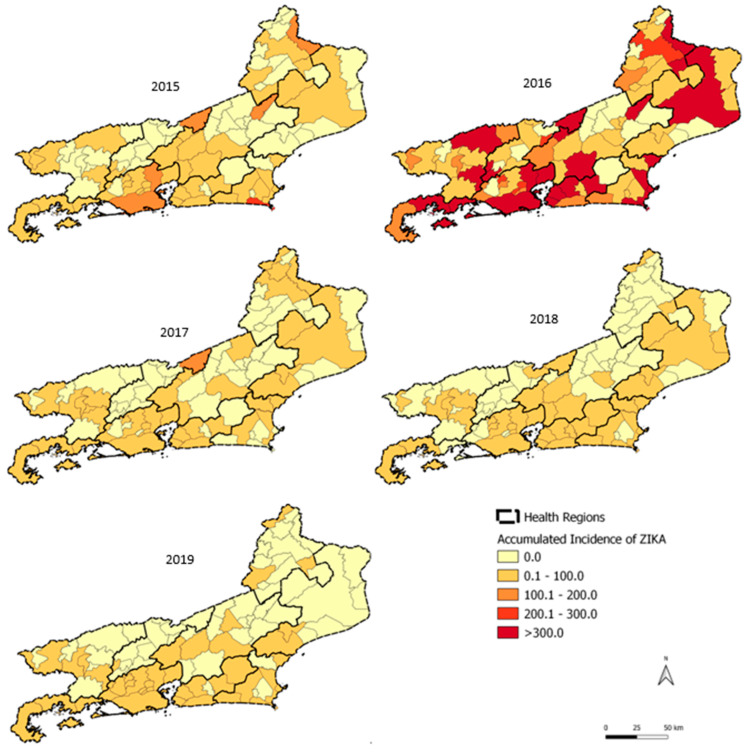
Zika by year of symptom onset in the state of Rio de Janeiro, 2015–2019.

**Figure 8:**
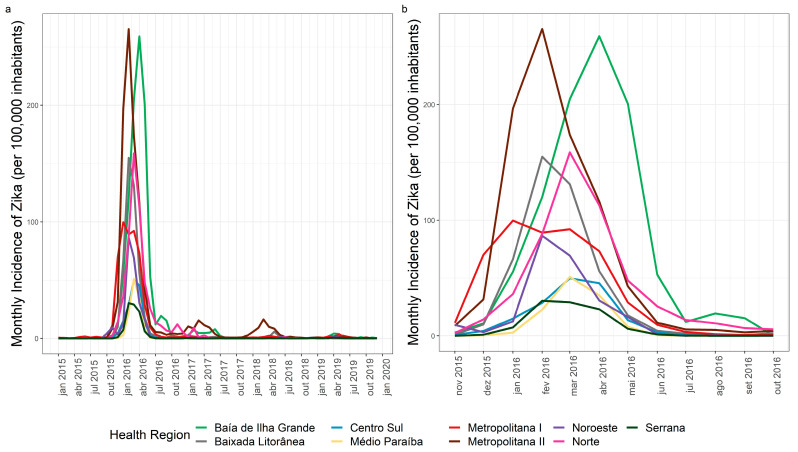
(**a**) Zika by month/year of symptom onset in Rio de Janeiro state, 2015–2019. (**b**) Zika by month/year of symptom onset in Rio de Janeiro state, November 2015–October 2016.

The Metropolitana II region, specifically the municipalities of Niterói, São Gonçalo, and Itaboraí, and Baía de Ilha Grande in the municipality of Angra dos Reis presented incidences above 300 cases/100,000 inhabitants (Figure 7). The highest monthly incidence for Zika in 2016 was recorded in the Metropolitana II region (265.4 cases/100,000 inhabitants) in February and in Baía de Ilha Grande (259.1 cases/100,000 inhabitants) in April (Figure 8a). Zika showed a heterogeneous pattern in its dispersion in each health region of the state. The Metropolitana I region reached the highest peak first, in January 2016. Subsequently, Baixada Litorânea, Metropolitana II, Noroeste, and Serrana regions peaked in February, and the Centro-Sul, Médio Paraíba, and Norte regions in March, followed by the Baía de Ilha Grande region in April 2016 (Figure 8b).

## 4. Discussion

We analyzed the spatial and temporal dynamics of the simultaneous circulation of dengue, chikungunya, and Zika in the entire state of Rio de Janeiro, considering its 92 municipalities distributed across nine health regions during the epidemic years from 2015 to 2019. Our results showed a heterogeneous distribution of the three viruses in time and space. In addition, some municipalities with higher population densities in each region had higher incidences and appeared to contribute to the dissemination and maintenance of these urban arboviruses. Two epidemics of dengue in consecutive years (2015 and 2016), one epidemic of Zika (2016), and one epidemic of chikungunya (2019) were detected in the state of Rio de Janeiro.

Temporal analysis showed that dengue and chikungunya presented later monthly incidence peaks (March to May) compared to that of Zika (January to March). Dengue was more dominant than the other two viruses, presenting high incidence rates in November and December 2015, which resulted in higher rates in the following year (2016). Dengue maintained high incidences during the five years of study in municipalities, unlike chikungunya and Zika. However, chikungunya was introduced into Rio de Janeiro in 2014, and one year later, the Zika virus arrived in the state, although previous studies have suggested the presence of Zika in the state since 2013 [11].

Despite the established transmission of Zika in 2015, since both the definition of notified cases and the instruments of notification were not defined or standardized and because it presents clinical signs similar to those of dengue, it is likely that some of the notified cases of Zika and chikungunya were misdiagnosed [22]. Many dengue cases were reported in 2015 and 2016 in Rio de Janeiro; however, a high percentage of laboratory-negative dengue cases was also confirmed, which were temporally related to Zika and chikungunya epidemics in the city [22].

Regarding the nine health regions of the Rio de Janeiro state, the Metropolitana I, Metropolitana II, Noroeste, and Norte regions presented higher chikungunya incidences, with municipalities showing medium to high average numbers since 2016 that extended each year until the epidemic reached all regions of the state in 2019. Zika presented a distinct pattern and caused an epidemic in 2016. Thus, regions recording increased incidences of the three diseases in their territories for long periods, especially when new arboviruses were introduced, should be considered municipal poles, as they initiated and sustained high transmission within their region.

In this study, distinct temporal behaviors were observed for each arbovirus. Dengue appeared to modulate the behavioral pattern of the other arboviruses, following the epidemic curves of both chikungunya and Zika. The peak in the epidemiological curves of dengue and chikungunya began in March and was sustained during April and May, whereas that of Zika was concentrated from January to March. Therefore, the peaks of dengue and chikungunya transmission curves in the epidemic period were later than those of Zika, a pattern similar to that found by Freitas et al. in the municipality of Rio de Janeiro [23].

Despite the fact that Brazil and its states have been developing actions to combat *Ae. aegypti* for over 70 years, including the use of larvicides, no success has been achieved in reducing or eliminating dengue epidemics or in preventing the entry of new arboviruses. Though not observed directly in this study, an intense and simultaneous occurrence of the three arboviruses occurred in 2016 in the city of Rio de Janeiro, as observed in another study resulting in the phenomenon of “triple epidemics” which was not observed in other years [23]. The results of this study indicate a biennial pattern, with two consecutive years of high transmission of dengue, chikungunya, and Zika, pointing to an increase in transmission in a given year with a subsequent epidemic in the following year. Dengue cases increased in 2015, already registering epidemics, which were even more intense in 2016. Chikungunya also increased cases in 2018, with a subsequent epidemic in 2019. Zika was introduced in the state of Rio de Janeiro in 2015 with an increase already seen in that same year, and in early 2016, the state recorded a Zika epidemic. In addition, we observed a decrease in chikungunya cases in 2016, concomitant with an increase in Zika cases, which has also been reported in other studies [18,23,24]. Thus, unlike Zika, chikungunya did not cause an epidemic in the state of Rio de Janeiro in the year following its introduction. One hypothesis is attributed to the entry of Zika, in which the same mosquito vector would not have been able to sustain the simultaneous transmission of two arboviruses [20].

Zika presented the lowest percentage (27%) of municipalities with annual cumulative incidences above 300 cases/100,000 inhabitants in its epidemic year, whereas dengue reached 59% and chikungunya included 41% of municipalities in the state with high annual cumulative incidences in their epidemic years.

Spatial analyses of the three studied arboviruses showed distinct behaviors among municipalities and health regions. Except for Baía de Ilha Grande and Médio Paraíba, the other regions presented higher curves of dengue cases in 2016 than in 2015, suggesting that the increase in incidence in 2015 caused a more intense transmission in 2016. This pattern was especially observed in the Norte, Noroeste, Baixada Litorânea, and Metropolitana II regions, which presented late peaks of dengue in 2015, specifically in July in the Norte and Noroeste regions, and in May in the Baixada Litorânea and Metropolitana II regions. In January 2016, all of these regions reached their highest monthly incidence rates for dengue: 127.5 in the Norte region, 1390.5 in Noroeste, 192.2 in Litorânea, and 157.7 in Metropolitana II.

Regarding chikungunya, the spatial and temporal analysis showed that in 2016, Metropolitana I was the only region that reached its highest monthly incidence rate in April, while the other regions of the state maintained very low monthly rates. In 2018, the Metropolitana II region had the highest number of chikungunya cases in April. In 2019, the disease reached all the regions of the state, suggesting a pattern of dissemination starting in large urban centers (Metropolitana I and II) and expanding to other areas of the state. This pattern was also observed for Zika, where the Metropolitana I region peaked in January 2016 with the highest incidence rate and Metropolitana II in February 2016, with the other regions peaking in different months starting in February 2016. In addition to the abundance of *Ae. aegypti* in the state (Epidemiological Report 002/2020, SES/RJ), the absence of a previously immunized population and high urban mobility, especially from large urban areas to the interior of the state, appear to have modulated the transmission profile of these two new arboviruses. Santos et al. [24] described the influence of population density and urban mobility in a spatiotemporal study of dengue in the city of Rio de Janeiro.

Previous studies have reinforced the high infestation of *Ae. aegypti* in the urban areas of Rio de Janeiro [10]. Therefore, in the presence of an entire susceptible population, the dissemination profile of a new arbovirus introduced in the state of Rio de Janeiro likely has a dispersion pattern strongly related to urban factors, with high primary transmission in large urban cities and subsequent dissemination to less densified areas. The presence and persistence of a higher risk of dengue occurrence in areas with high urban growth and without adequate infrastructure reaffirms this scenario, which occurs in all health regions of the state [22,24,25]. The uncontrolled growth of the urban population associated with poor sanitation and housing conditions in large urban centers provides favorable ecological conditions for dengue virus transmission. Therefore, localities with a higher urban population may present greater incidences of dengue, as described in a Rio de Janeiro state study during the 2002 epidemic, where the urban population proportion showed a direct association with the incidence of dengue [26].

A better understanding of the behavior of these arboviruses will contribute to the implementation of prevention and control measures that minimize their effects. The reasons for successful dissemination of arboviruses are complex. Different surveys indicate certain factors, such as agent pathogenicity, *Aedes* infestation, deforestation, disorderly occupation of urban areas, poor sanitation, and climatic, demographic, and social changes as the main elements facilitating this process. Moreover, it is known from chikungunya virus studies that the increase in the movement of people between countries is considered one of the main determinants for the introduction of these arboviruses [2,27,28,29]. A better understanding of the phenomena involved in the transmission of arboviruses in each location can contribute to modelling studies that support prevention policies [30,31].

Co-circulation of the three arboviruses in the state and heterogeneous spatiotemporal distribution was observed for each arbovirus. However, there is an urban pattern of dissemination of chikungunya and Zika, beginning with a high occurrence in large cities followed by expansion to cities with lower demographic densities [32,33]. This study can contribute to a better understanding of the underlying factors of each region and/or municipality, thereby allowing the subsidization of surveillance and control actions in each territory of the state of Rio de Janeiro.

## Figures and Tables

**Table 1:** Dengue, chikungunya, and Zika by year of symptom onset, Rio de Janeiro state, 2015–2019.

Cumulative State Incidence by Year															
	**DENGUE**					**CHIKUNGUNYA**					**ZIKA**				
	2015	2016	2017	2018	2019	2015	2016	2017	2018	2019	2015	2016	2017	2018	2019
	382.6	509.5	64.0	85.0	182.7	0.5	105.1	27.3	230.7	492.8	62.4	430.9	15.3	14.5	8.9
**92 Municipalities**															
Average	806.6	1102.9	59.5	72.9	207.4	0.3	22.7	17.1	188.9	580.0	15.7	316.9	6.4	6.3	4.1
Standard Deviation	1463.1	1694.4	126.7	144.8	381.9	1.0	43.6	48.0	455.7	1015.3	37.4	796.9	22.0	15.2	12.4
Median	206.1	454.4	17.6	25.9	76.9	0.0	5.0	3.0	17.7	201.2	0.6	47.5	0.0	0.0	0.0
Minimum	0.0	6.3	0.0	0.0	0.0	0.0	0.0	0.0	0.0	0.0	0.0	0.0	0.0	0.0	0.0
Maximum	9153.6	9887.5	1019.1	1143.7	2677.0	7.4	258.9	326.0	2588.7	5995.6	216.5	6788.2	174.5	76.4	83.7
**Number of municipalities with incidence > 300 cases/100.000 inhabitants**															
	39	54	3	4	16	0	0	1	12	38	0	25	0	0	0

## Data Availability

Not applicable.
